# Medical Ethics

**Published:** 1847-02

**Authors:** 


*Medical Ethics.*—We copy the following article from the last number of the Bulletin of Medical Science. The sentiments contained in the quotation from the Lancet, and in Dr. Bell's commentaries will be deemed sound and wholesome by every high-minded practitioner. We are glad to see the subject of formal instruction in Medical Ethics suggested. It has often presented itself to our reflections, and we trust it will receive the favorable consideration of medical teachers.—*Ed. Buff. Jour.*

*Medical Ethics.*—In the propriety and advantage of the subjoined recommendation, from tho London Lancet, we fully concur. Were the subject of Medical Ethics appreciated as it ought to be, and made an integral part of education, Professors, while teaching it, would avoid the exhibitions of caprice, spleen and jealousy, which, in many instances, they do not attempt even to veil, under a fillagree work of boastful promises and sophistical advice to their students; while the latter would be indoctrinated with sound principles, to guide them in their future intercourse with their professional brethren and the general community, so far as that intercourse grows out of their medical position and duties. If the ethical obligations of medical men, as such, were properly understood and felt, both the profession and the world at large would be saved those discreditable exhibitions of corporate and official intolerance and selfishness, and of individual vanity and scheming, which are now so common, that bad precedent and bad example threaten to usurp the right, and make the expediency of the hour the rule of conduct.

It may be alledged, that all the duties and obligations directed by Medical Ethics are included, and, impliedly, taught in the precepts of morality and religion, and that if these latter be fully understood and observed, we need not trouble ourselves about the former. To this we would reply, that, although it is convenient to aid the memory, and to fix the attention by condensed propositions in morals and religion, yet every teacher, yea every intelligent being knows the necessity, for his better information, of extended commentary, and, also, of special caution and direction under all the circumstances in which he may be placed. Men of every class and calling, in a civilized community, profess to be governed by the same moral code; but, yet, how different the modes in which its details are worked out and its observances complied with, according to the temptations and risks of collision and breach in various branches of occupation and business. All right-minded men will refrain from what they believe to be positive wrong, and will refuse to give it their sanction and support; but, on particular occasions, unexpected circumstances and cases not ruled by their familiar precedents, occur, and, without meaning wrong, some will be found to commit it, or by omission of a direct duty, to allow its force, through want of check and rebuke.

We can attribute only to ignorance of medical ethics much of the countenance which quackery has received from physicians of eminence and admitted disinterestedness, so far, at least, as an absence of any pecuniary gain or personal advantage was concerned. Sound logic, ought, it is true, to have restrained them from giving support of this kind to men, who measured by its standard, could not be supposed, by their known ignorance and want of opportunity to make improvements, still less discovery, to be entitled to any countenance, or consideration, even though they should not assume the garb of mystery, and lay claim to special privileges in vending their panaceas, pills, &c. But we can understand, still, how a medical man might, on the plea of trying everything that offers, by the test of *lædentia* and *juvantia*, and the known uncertainty of therapeutics, think it justificable to give the ignorant charlatan a chance of ascertaiaing the virtues of his nostrum,—did not medical ethics, exhibiting the moral bearings of the subject, show that there was knavery, craft, and cunning, as well as ignorance, in this pretence of discovery; and that the whole history of the nostrum is one of deception and falsehood—injurious to the community at large still more than to the profession itself.

From the recommendation and use of quack remedies to a participation in their manufacture and sale, the step is short and easily made : an infringement on the code of medical ethics, practiced by the professor and aged physician, in the first case giving example and encouragement for a still farther breach on the part of the young, the experienced, and, it may be, the needy, in the second case: The people seeing quacks and quackery thus fostered by physicians, follow the example; and, believing that chance rules the hour, make selections for themselves. Need we wonder, then, that clergymen, judges, lawyers, and legislators, as well as the more numerous crowd, are ready to append their name to empirical pretensions of all kinds; they but follow in the wake of members sometimes the *elite*—save the mark!—of the medical profession, who are supposed to be the conservators of the health of their fellow citizens.

We might extend the argument, while increasing the examples of the bearing of medical ethics; but our purpose for the present, is answered, by having shown, that a special code is necessary for our guidance under circumstances and in situations of professional life, which are not met, for immediate action and practical purposes, by the general obligation of duty to our neighbor. In reverting to the implied advice, at the beginning of these remarks of ours, that Medical Ethics should constitute an integral part of medical education, we may be allowed to express a hope, that, ere long, a volume, consisting of a recognized code, and suitable commentary, of Medical Deontology will figure on the cards of our medical schools, as regularly as the favored text-book of each professor. We would go a step farther, and recommend that such a volume should be put into the hands of a successful candidate for a degree, by the Dean, in the name of the assembled Faculty.

"We have a right, in laboring for the good of the whole profession, to claim the co-operation of all. More especially do we claim the aid of the legitimate professors and teachers of the various departments of medicine throughout the kingdom. They have, to a great extent, the power of moulding the opinions and feelings of the next medical generation. As "the child is father to the man," so is the student the parent to the future practitioner. We have labored to show that many of the mischiefs of quackery might be cured or prevented by a sound and orthodox state of professional opinion. To produce a wholesome and catholic feeling of respect for the honor of the profession, and an emulation in the minds of young men so to conduct themselves, both in studentship and in practice, as to uphold and reflect honor upon the faculty to which they belong, is, at the present time, the duty of every public teacher. There ought undoubtedly to be, considering the state of the profession, a special professor of Medical Ethics in every great school of medicine in the kingdom. In recent years, there has been a disposition to found theological exhibitions and religious prizes in the medical schools; students have had held out to them, in some instances, very liberal inducements to attend prayers regularly, and to mix up the study of divinity with their anatomical dissections. With the good intention of all this we will not quarrel. But in the first instance, we would certainly have the ethics of professional life made the subject of teaching *within* our schools. Theology already has its appropiate channels, and we repeat the remark, that *within the schools* the young professional man should be taught to think of his duties towards his professional brethren, as his nearest neighbors in his passage through life. We earnestly commend this subject of thought to the different public teachers in medicine. As there is not at present, in any medical college, a chair of medical ethics, we trust each individual teacher, on the many occasions that must offer in every class, will do something to supply the deficiency. That the contention of the opposing elements, QUACKERY and sound MEDICAL ETHICS, will become of increased interest to the entire faculty, we are fully persuaded; and of the result—the overthrow of the Hydra from which medicine suffers—there can be little doubt, if the profession be but true to itself.

